# Synchronicity of the Gulf Stream path downstream of Cape Hatteras and the region of maximum wind stress curl

**DOI:** 10.1038/s41598-024-68461-0

**Published:** 2024-08-09

**Authors:** Ian Gifford, Avijit Gangopadhyay, Magdalena Andres, Hilde Oliver, Glen Gawarkiewicz, Adrienne Silver

**Affiliations:** 1 Department of Physics, University of Massachusetts at Dartmouth, Dartmouth, MA 02747 USA; 2 School for Marine Science and Technology, University of Massachusetts at Dartmouth, Dartmouth, MA 02747 USA; 3 Woods Hole Oceanographic Institution, Woods Hole, MA 02543 USA

**Keywords:** Climate sciences, Ocean sciences

## Abstract

The Gulf Stream, a major ocean current in the North Atlantic ocean is a key component in the global redistribution of heat and is important for marine ecosystems. Based on 27 years (1993–2019) of wind reanalysis and satellite altimetry measurements, we present observational evidence that the path of this freely meandering jet after its separation from the continental slope at Cape Hatteras, aligns with the region of maximum cyclonic vorticity of the wind stress field known as the positive vorticity pool. This synchronicity between the wind stress curl maximum region and the Gulf Stream path is observed at multiple time-scales ranging from months to decades, spanning a distance of 1500 km between 70 and 55W. The wind stress curl in the positive vorticity pool is estimated to drive persistent upward vertical velocities ranging from 5 to 17 cm day^−1^ over its ~ 400,000 km^2^ area; this upwelling may supply a steady source of deep nutrients to the Slope Sea region, and can explain as much as a quarter of estimated primary productivity there.

## Introduction

The Gulf Stream (GS) is well recognized as the northward-flowing western boundary current of the anticyclonic North Atlantic subtropical gyre. At Cape Hatteras, North Carolina the GS separates from the continental margin and becomes a free jet flowing east-northeastward. Gulf Stream path variability over the 2500-km distance downstream of Cape Hatteras has impacts that span fisheries responses^1^ to atmospheric events^2^ and the GS path is often interpreted as an indicator of climate-related changes in the Atlantic Meridional Overturning Circulation (AMOC,^3,4^). Recent changes in the northwest Atlantic water properties and ecosystems have been linked to the variations of the GS path and to Warm and Cold Core Rings (WCRs, CCRs) shed from the meandering current^5–14^. Andres^5^ reported that the destabilization point of the GS, the point at which the GS transitions from a stable to a vigorously meandering current, moved westward, closer to Cape Hatteras, in the late 1990s. Additionally, a recent study by Wang et al.^15^ reported that the Gulf Stream has moved northward west of the New England Seamount Chain (NESC; ~ 65° W) in recent years, while moving southward east of the NESC, in agreement with previous studies^16–18^. An observation-based ring census shows that the annual number of WCR formations underwent a regime change around 2000, nearly doubling from an average of 18 rings shed per year (1980–1999) to 33 per year (2000–2017)^7^, while the CCR formations did not show such a change^13^.

Many previous studies have tried to capture the GS path variability with a simple path index. These indices apply statistical techniques to GS surface characteristics, related to the location of the current maximum or the sea surface temperature gradients^19^, or to GS subsurface markers, e.g., a particular isotherm at a particular depth^20^. These surface or subsurface measurements at various discrete longitudes are then combined to obtain a single path-integrated index of the GS position at monthly or annual time scales. Chi et al.^21^ summarized a number of these different GS indices. However, the complexities associated with along-stream longitudinal variability of the often multi-valued GS path behavior—such as the different periodicities of the path to the west and east of the NESC^16^ or the asymmetrical movement of the GS east and west of 65° W^15^—are obscured in such path-integrated index representations of the GS position. Furthermore, using path-integrated indices makes it hard to identify the driver(s) of GS path variability such as those indicated above. This is borne out by different studies which indicate conflicting correlations between the GS path and the North Atlantic Oscillation (NAO, an atmospheric index related to the wind and pressure fields) over different time periods and at different time scales^17,19,20,22–25^, possibly due to different representations of the GS path from different single integrated indices spanning different longitudinal domains in the different studies.

Among the potential factors affecting the mean GS path and its variability are the overlying mean and time-varying wind-stress curl fields. The basin-wide wind stress curl over the subtropical North Atlantic and the associated westward propagating Rossby waves have been linked to the formation of the western boundary current (Florida Current), its northward transport along the US eastern seaboard between Florida and North Carolina, and its separation latitude near Cape Hatteras^26–29^. The NAO has been linked with the transport and north-south movement of the Gulf Stream path after separation [Refs.^19,22,30^ and references therein]. In these studies, the dynamical impact of the wind stress curl during different phases of the NAO was cited as the primary factor driving the observed responses of the GS path at and beyond the separation latitude.

In the North Pacific Ocean, decadal shifts of the Kuroshio Extension, the North Pacific analog to the separated Gulf Stream, are associated with a weak (strong) transport and an unstable (stable) meandering configuration^31^. These opposing phases have been linked to the basin-wide wind-stress curl, which forces negative (positive) sea surface height (SSH) anomalies that propagate westward in the form of Rossby waves during negative (positive) phases of the North Pacific Gyre Oscillation. Furthermore, it was recently shown that the strong and stable state of the Kuroshio Extension is also associated with a strong southern recirculation gyre^32^. Wind stress curl anomalies over the North Atlantic may similarly affect the state (stable versus unstable path) of the GS as speculated by Kelly et al.^33^. The sensitivity of the GS path to cyclonic wind stress curl was investigated by Gangopadhyay and Chao^34^ using a 1/6-degree numerical model with climatological^35^ and operational (ECMWF)^36^ wind forcing. It was found that the SST-based GS north wall followed the wind-stress curl maximum over a period of 4 years (1983–1986) in the model forced by the operational fields, but not in the model forced with the coarse climatology (see Gangopadhyay and Chao^34^, their Fig. 2). Untangling the various effects responsible for the interannual variability of the GS path downstream of the separation remains an area of active research and motivates this study.

This study investigates the relationship between GS path downstream of Cape Hatteras, and the overlying wind stress curl field. There are conflicting conceptual models about whether the Gulf Stream path after separating from coast follows the zero wind stress curl (as elucidated by linear theories) or the maximum wind stress curl north of the zero curl (as established from early non-linear numerical experiments). Our study aims to address these conflicting conceptual models from a purely observational perspective from the 27-years of satellite data of GS path and atmospheric wind field reanalysis. Details of the data sets and methods used to characterize the GS path and the wind stress curl (WSC) field are given in the Supplementary Information (SI). The following analysis of twenty-seven years (1993–2019) of a wind reanalysis product and satellite SSH data demonstrates that the path of the freely meandering GS between 70° and 55° W is synchronous with the region of maximum cyclonic vorticity of the wind stress field (known as the positive vorticity pool). This study also examines the interannual variability in this positive vorticity pool and the implications of this variability on upwelling and ecosystem responses and concludes with discussion of possible future theoretical developments based on the observed correlations.

## Observation: wind stress curl spatial pattern and location of the GS free jet

**Figure 1:**
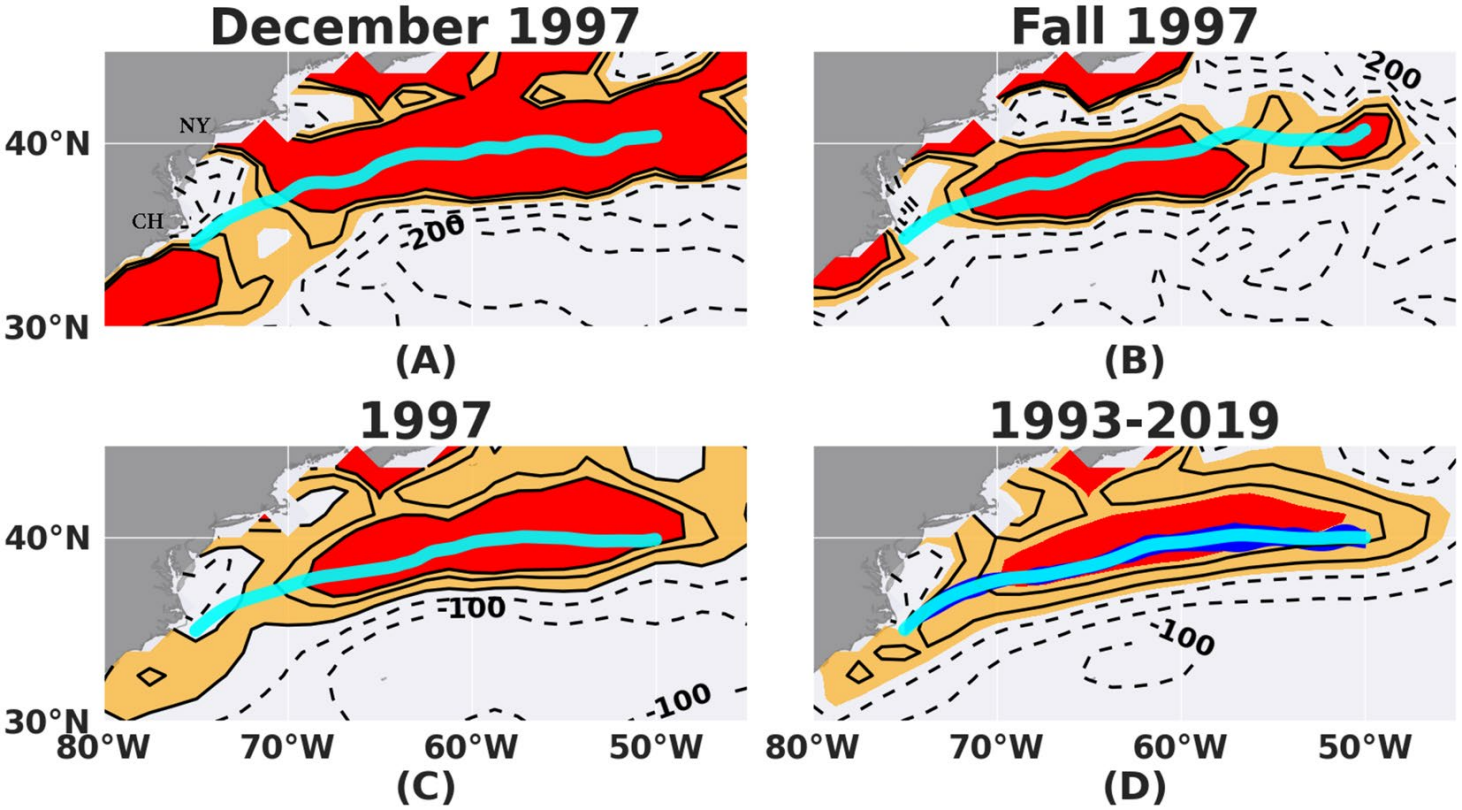
Multi-scale synchronicity of the region of maximum wind stress curl and the GS path. (**A**) Monthly example (Dec, 1997), (**B**) Seasonal example (Fall: OND, 1997), (**C**) Annual example (1997), and (**D**) 27-year (1993–2019) averaged WSC (contours) superimposed with corresponding averaged GS path (cyan line) from altimetry over the domain of 80° W to 45° W and 30° N to 45° N. Curl amplitude values are shown in contours of Pa/m × 10^–9^. The monthly, seasonal and annual fields are shown for the year 1997 as an example. The east coast of USA is shown in gray with Cape Hatteras (CH) near 75° W, 35° N and the city of New York (NY) near 74° W, 41° N marked for reference in panel (**A**). See also Fig. S1 and movie S1 in Supplementary Information (SI) for all individual years from 1993 through 2019**.** All monthly equivalents are shown in movie S2 in the SI. Shaded (orange and red) regions in all panels are regions of positive vorticity with the ‘positive vorticity pool’ (see text) shaded red (≥ 80 Pa/m × 10^–9^). Yellow is < 80 Pa/m × 10^–9^. Dashed contours denote negative curl. The blue ‘envelope’ in (D) indicates the latitudinal spread of the 1993–2019 annual GS path means calculated at each 0.1° longitude bin.

To investigate the relationship between the wind and the GS, the co-variability of the WSC field over the domain 30–45° N, 80–45° W from the Japanese 55-year Reanalysis (JRA-55) is compared with variability in the path of the free GS jet from 75° W to 50° W, as inferred from the altimeter SSH data. See Materials and Methods within the Supplementary Information for the determination of the wind stress curl field from JRA-55 and for identification of the GS axis path based on the method of Andres^5^. Daily fields of both WSC and GS path are averaged over various time-scales (monthly, seasonal, annual, and interannual) and these averaged fields are then compared (e.g., Fig. 1). The synoptic variability of the wind stress curl of individual month and season for each year with the relevant GS path during 1993–2019 superimposed is presented in Figs. S1, S2 and S3, and movies S1 and S2; and the wind stress curl data are provided in Gifford et al.^37^.

As evident in Fig. 1, the WSC field in the northwestern North Atlantic generally has two extrema associated with the mid-latitude westerlies– one negative to the south of a zero-curl line and one positive to the north of the zero-curl line. For the year shown, 1997, the location of the axis of the GS downstream of Cape Hatteras lies within the region of positive maximum wind stress curl at all time-scales examined—monthly (Fig. 1A), seasonal (Fig. 1B), and annual (Fig. 1C). This synchronicity between the GS and the WCS maximum region is also evident in the 27-year average fields (Fig. 1D). Also, Fig. S1 confirms this synchronous pattern between the WSC maximum region and the annually-averaged GS paths for each of the 27 years (1993–2019) examined here. Furthermore, Figs. S2 and S3 provide the average monthly and seasonal patterns of the curl and the corresponding GS paths, supporting their synchronicity at these timescales.

This qualitative relationship observed between the positive WSC maximum region (i.e., the ‘positive vorticity pool’) and the path of the free GS jet provides the motivation to determine a way to quantify changes in the strength and extent of the region of maximum wind stress curl within which vorticity is strongly positive (cyclonic). Identification of the ‘positive vorticity pool’ allows for characterization of interannual variability in the curl maximum amplitudes and patterns and provides a means to quantify temporal changes including the area it occupies, as well as the relationship between this positive vorticity region and the free GS jet.

**Figure 2:**
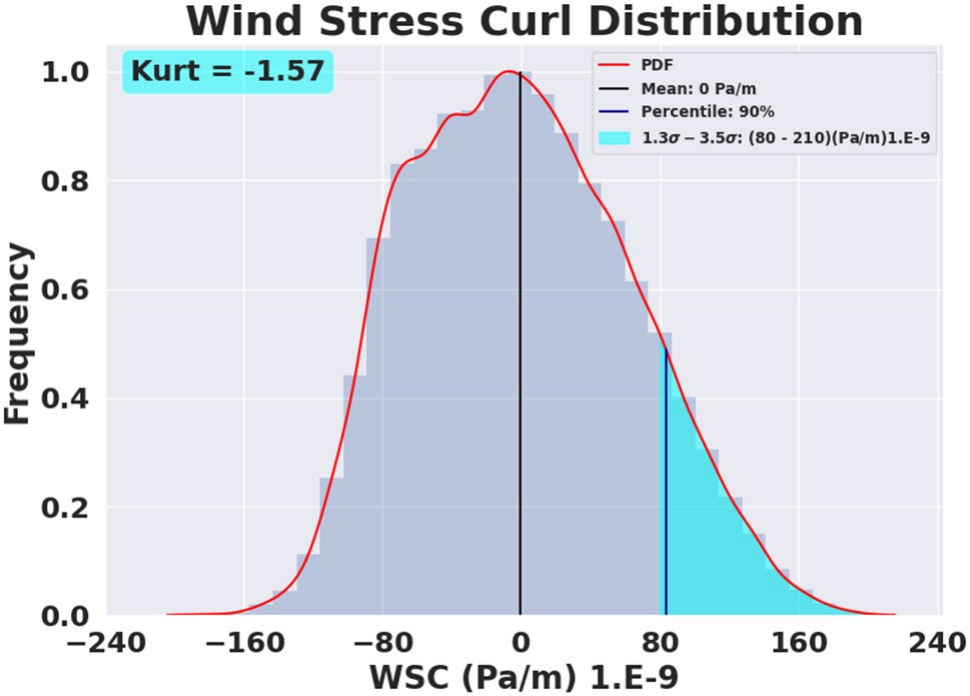
Distribution of wind stress curl over the North Atlantic (30–45° N, 80–45° W). This distribution is based on 27 years of wind stress data. See Methods for details. The horizontal axis extends from the most negative WSC value to the most positive WSC value. The cyan region is defined as the region of maximum wind stress curl or the positive vorticity pool. The mean is the black line located at 0 Pa/m and the blue line demonstrates the lower bound of the observed maximum as the 90 percentile.

As a first step to quantify interannual variability in the positive vorticity pool, the distribution of the daily WSC within the domain over the entire 27 year period is examined to evaluate the relative frequency of occurrence of different curl values within the region. The methodology to generate this wind stress curl distribution is presented in Materials and Methods within the SI and the distribution is shown in Fig. 2. Based on this distribution, a positive curl ‘threshold’ is chosen such that it exists in every year (1993–2019) within the region of interest and, by definition, it bounds each year’s annually-averaged positive (cyclonic) vorticity pool (i.e., the red shaded region in Fig. 1 and in Figs. S1 and S2).

Based on the curl distribution in Fig. 2, a threshold is chosen at 80 × 10^–9^ Pa/m (which is the value at 1.3 times the standard deviation, σ, of the distribution); this threshold defines a wind stress curl value above which the maximum region (i.e., the positive vorticity pool) lies for all the individual years. The region of the WSC maximum is then defined by the area occupied by values that fall within the observed range bounded by 1.3σ to 3.5σ above the mean of the field (cyan shaded region in Fig. 2). The 1.3σ limit is chosen as it is very close to the 90^th^ percentile of the observed curl range indicating the robustness of this choice. An excess kurtosis of − 1.57 indicates the presence of outliers in the distribution. This area of the positive vorticity pool (i.e., within which the WSC exceeds the threshold value) provides a metric to determine the relationship between WSC maximum and the path of the free GS jet.

## Results: variability of the wind stress curl parameters

**Figure 3:**
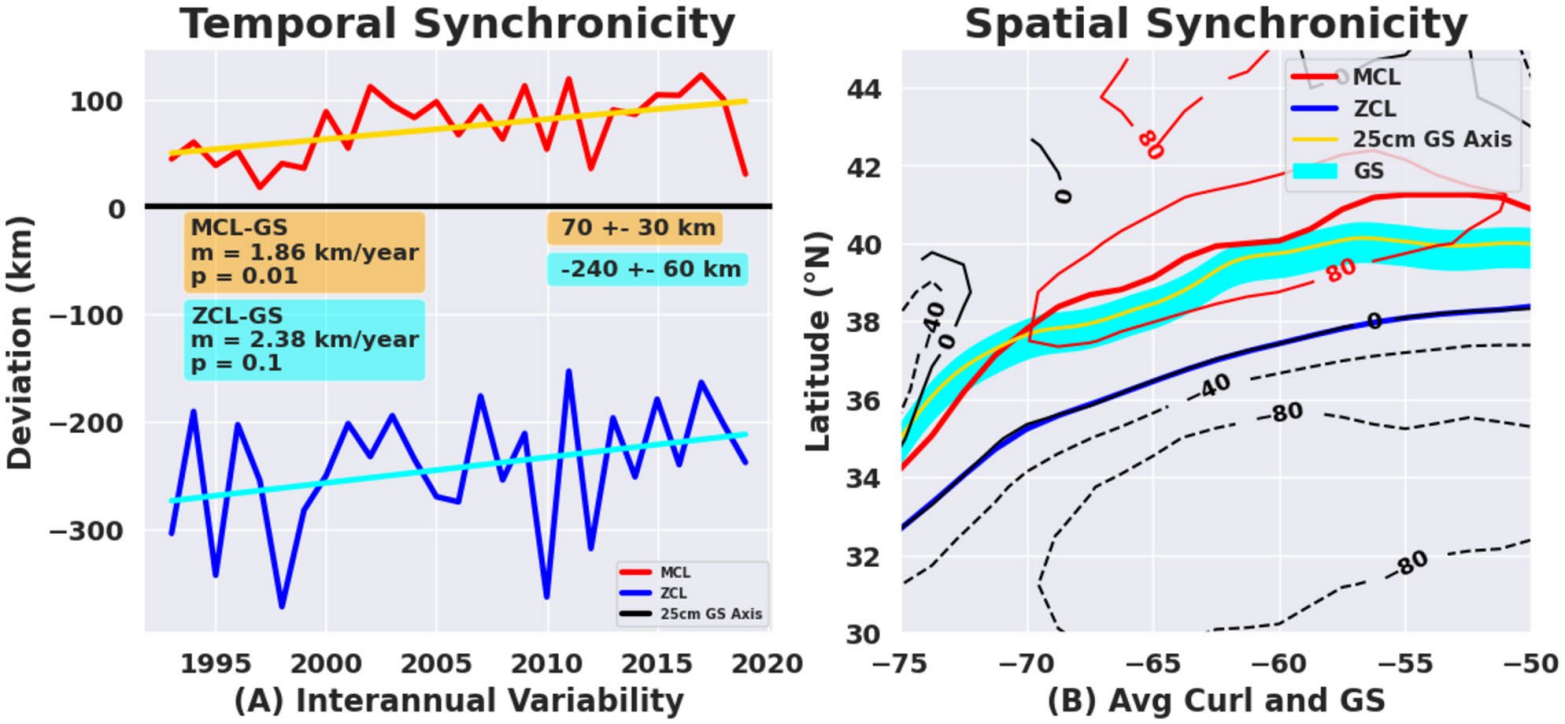
Temporal and spatial synchronicity of the GS path with the wind stress curl. (**A**) Temporal synchronicity with slow interannual variation over the 27-year study period**.** The average southward (northward) deviation from the GS axis to the zero curl line (ZCL, blue) and maximum curl line (MCL, red). The GS axis is situated at the y = 0 line, with the MCL to the north (positive) and ZCL to the south (negative) of this. (**B**) Spatial synchronicity of the maximum curl region (bordered by the red thin line) with the Gulf Stream (cyan shaded path with the GS axis in yellow) over the 27-year period. The maximum curl line is the solid red line over the positive vorticity pool. The zero curl line is the blue line to the south, which separates the negative wind stress curl region from the positive vorticity to the north where wind-driven upwelling is expected. Similar maps for the annual and monthly averages are shown in the Movies S1 and S2, which highlight the multiscale nature of this region-wise synchronicity.

**Figure 4:**
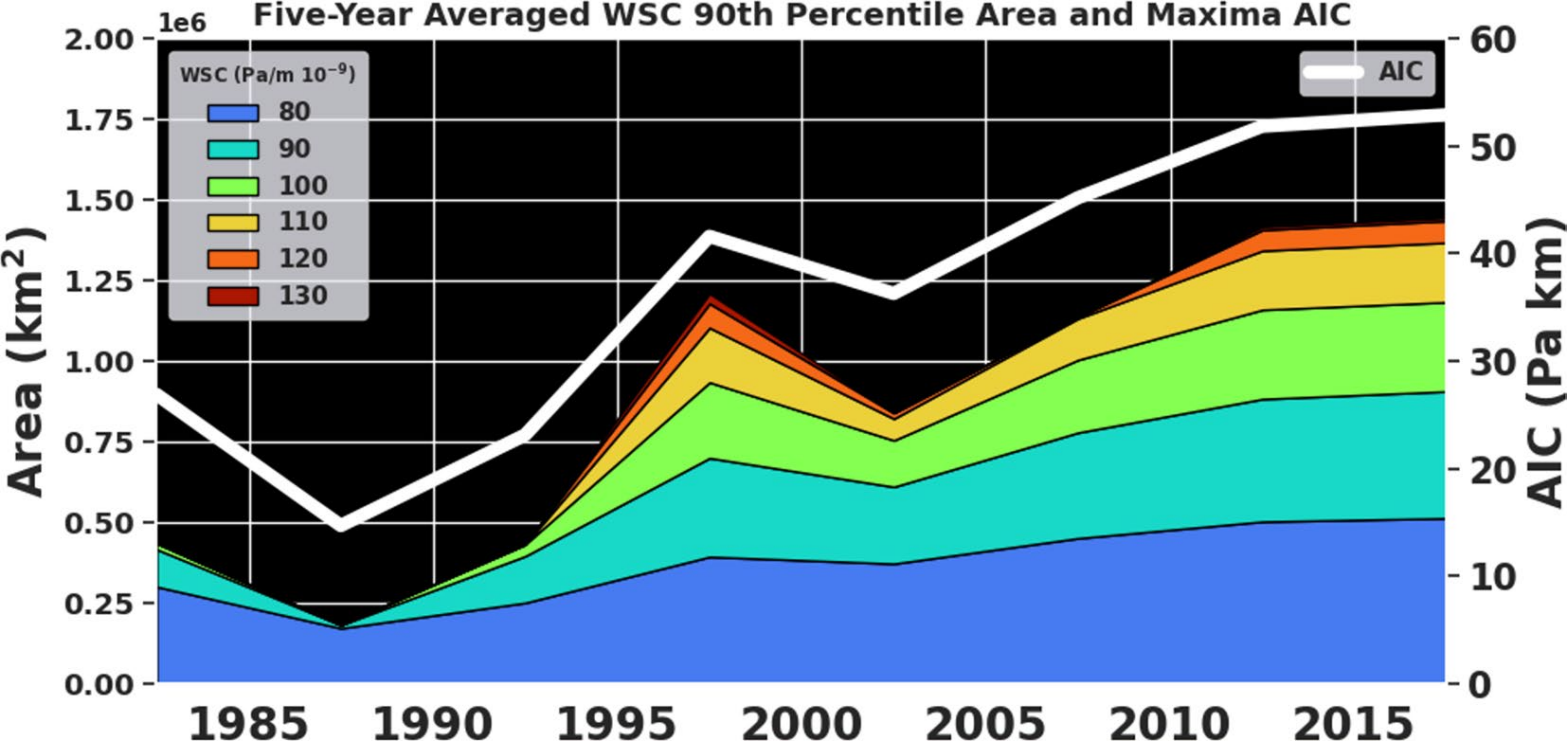
Temporal variability of the area of the positive vorticity pool region and the area integrated curl over this region. Five-year averaged area of WSC maximum broken down by its major contributory ranges of curl (see Gifford^41^ for contributions from specific maximum ranges). Units are in km^2^ for the area wedges. Note how the high magnitude curl areas have been increasing over the recent pentads. Five-year averaged area integrated curl, (see Gifford^41^ for values) are depicted in the solid white line, whose units are in Pa km and shown along the right axis.

As discussed above, the Gulf Stream path is a multi-valued curve in space and the wind-stress curl is a two-dimensional field, which for our purposes is best represented by the spatial extent (surface area) and the area-averaged value (amplitude) of the curl within a fixed-value contour such that the amplitude of curl within that area varies in different years. Thus, we choose to create the MCL (maximum curl line) and ZCL (zero curl line) metrices along with a smoother version of the Gulf Stream path between 75 and 50W, and then cast the problem with a proximity analysis as measured by ‘absolute deviation’ between these curves in space and time as presented throughout the analysis (Figs. 3 and 4 below).

### The maximum curl line (MCL) and the zero curl line (ZCL)

Two contour lines are chosen to delineate and characterize the mean wind field – one marks the zero of the wind stress curl and the other outlines the area encompassed by the threshold contour (80 × 10^–9^ Pa/m) of curl, or the region of the positive vorticity pool. These contour lines are obtained from the annual curl fields mapped at 0.1° intervals (see “Materials and methods” within SI). From this we establish two separate metrics for determining the statistical significance of co-variability in the observed patterns of GS path and winds: the annual mean deviation (absolute distance) between the GS path and the zero wind stress curl line (ZCL) and the annual mean deviation of the GS path to the maximum wind stress curl line (MCL) within the region of the positive vorticity pool. Both of these are depicted in Fig. 3A,B. The ZCL is used to compare and contrast the colocation of the GS path with the MCL. The ZCL is known to lie south of the GS path after separation (see Talley^38^—their Figs. S9.1 and S9.3; see also Seidov et al.^39^—their Figs. 2 and 3).

The slow interannual variation in the spatial synchronicity of the GS path and the region of positive vorticity pool (quantified by the location of the MCL) is presented in Fig. 3A and is compared here with the deviation of the stream path from the ZCL. On average (zonally-averaged over the domain), for the 27-year record, the annually-averaged ZCL is 240 km ± 60 km south of the GS jet axis (over the longitudinal domain considered, 70–55° W), while the MCL is 70 km ± 30 km north of the GS axis (Fig. 3A). Note that the typical width of the GS is 50 km to the north and 100 km to the south of the jet axis where the maximum near surface geostrophic velocity is found^40^ (see also Chi et al.^21^  -- their Fig. 1). This indicates that the maximum curl region is generally directly over the ~ 150-km wide GS current between 70° W and 55° W.

The wind field and its position relative to the GS have undergone low-frequency changes over the 27-year period examined here. The MCL has shifted northward relative to the GS path by about 50 km, and the ZCL has also shifted northward (closer to the GS) by about 64 km (Fig. 3A, trend lines). While the northward trend of MCL shift is significant at 95% level, that of ZCL shift is significant only at 90% level. The variability of the ZCL shift (± 60 km) is almost twice than the variability of the MCL (± 30 km), indicating the robustness of the proximity of the GS to the MCL compared to being close to ZCL. A recent study by Seidov et al.^39^ shows the positions of the zero lines and their relative southward separations from the GS North Wall locations of almost 200 km, which agrees with our observations.

To examine spatial patterns in the GS path and the wind field, and how they covary, the longitudinal variations of distances of the MCL and ZCL from the GS are examined at different time-scales (monthly, annually and over the whole 27-year period). The spatial synchronicity between the GS and the MCL region for the whole period is presented in Fig. 3B. The yearly and monthly variations of this spatial synchronicity are presented in the Movies S1 and S2 (SI). The GS passes through the region of maximum wind stress curl in all of the 27 years. Interestingly, the distance between the MCL and the ZCL is minimum near Cape Hatteras where the GS first separates from the western boundary and then increases eastward until about 50° W, after which the distance decreases again. This was also observed by Seidov et al.^39^ (see their Figs. 3 and 4). The amplitude of the maximum of the wind stress curl also increases eastward, reaching a maximum around 65° W and remaining high until about 58° W (Fig. 3b). East of this region, the amplitude decreases and the contours close to form the eastern boundary of the positive vorticity pool at around 50° W. For details, see Gifford^41^.

### Changes of the wind curl maximum region (positive vorticity pool) over 1980–2019

Next, we characterize changes in the area of the wind stress curl maximum or the ‘positive vorticity pool’ region and changes in the area-integrated curl (AIC) within this region over the study period (Fig. 4). Overall, the area and intensity of the maximum has increased over the GS region in the recent decades.

Therefore, larger portions of ocean surface area have been exposed to the WSC maxima (i.e., the positive vorticity pools) in recent years. Furthermore, contributions from larger WSC values (≥ 120 × 10^–9^ Pa/m) become prevalent in the 1995–1999 pentad and persist beyond this pentad, indicating possible changes in the forcing and upwelling within the free jet. The pentad spanning 2015–2019 shows the largest AIC value (~ 50 Pa km) over the temporal domain considered here.

Aside from changes in the spatial extent (area) of the WSC maximum, the interannual variability of the center of mass of the curl maximum (defined by the region bounded by 80 × 10^–9^ Pa/m contour) is also changing. Movement of the center of mass (COM) quantifies local changes in the position of the positive vorticity pool that may also result in changes in the region of strong upwelling. Upwelling-favorable wind stress curl has been documented to drive the upward transport of nutrient-rich deeper waters into the sunlit upper ocean, supporting elevated primary productivity near the coast^43–45^.

**Figure 5:**
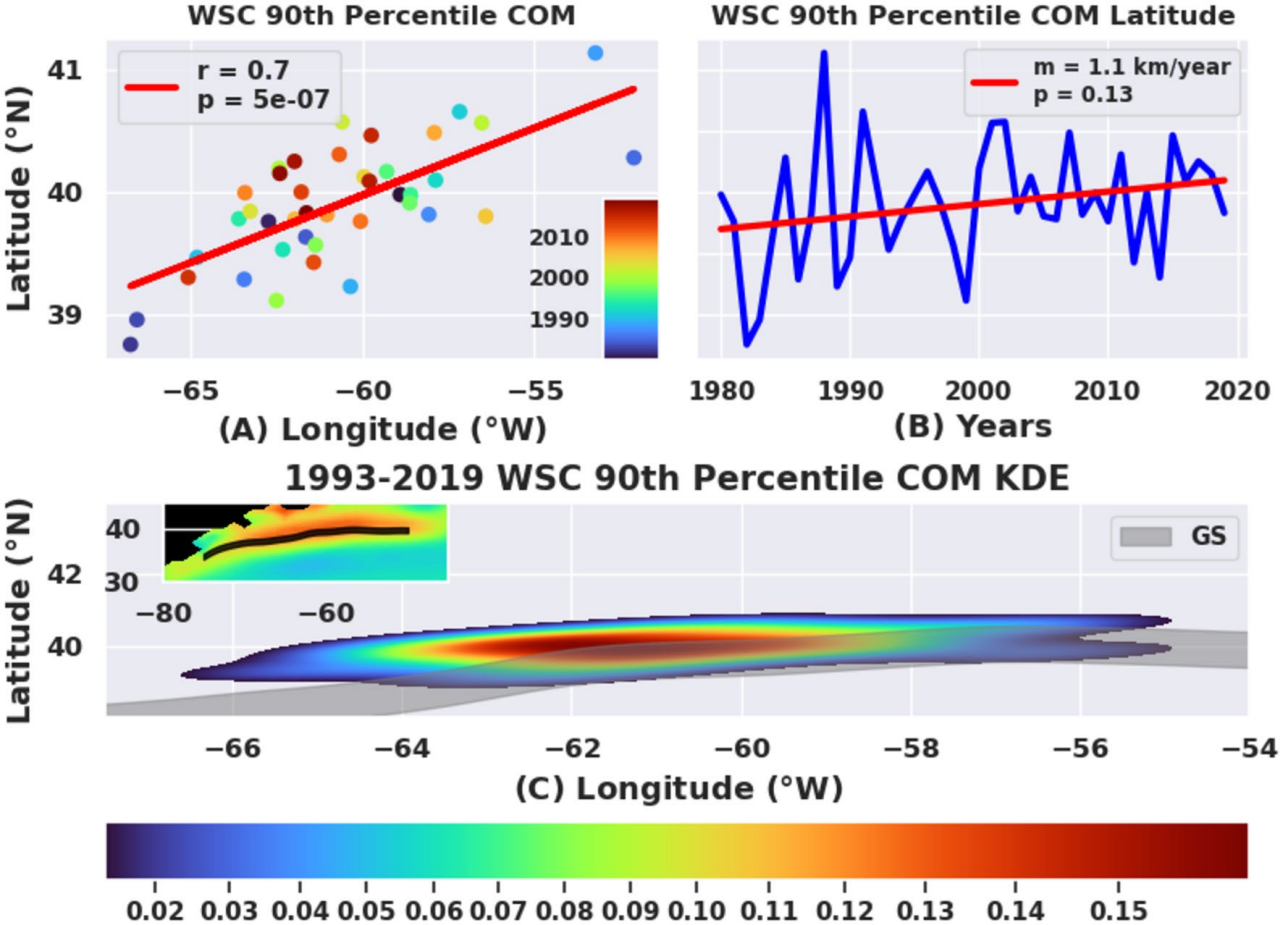
Spatial and temporal behavior of the center of mass (COM) of the positive vorticity pool. (**A**) The positive vorticity pool’s center of mass (COM) longitude against latitude over forty years (1980–2019). (**B**) Annually averaged COM latitude from 1980 to 2019 (blue) with line of best fit (red) showing possible northward movement of the COM during this period (p = 0.13). (**C**) A kernel density estimation (KDE) map of the distribution of COM location of a region bounded by the 90th percentile of the wind stress curl. The COM has been converging to a small region around 61°–62° W primarily due to recent years which are shown in red/orange dots in panel (**A**).

Figure 5A demonstrates a coupling (see the strong linear fit with r = 0.7) between the COM longitude and COM latitude, in that the COM is moving along a line that is not purely zonal or meridional. Furthermore, this non-unity correlation or the deviations from this line might be associated with the change of shape, size and amplitude of the positive vorticity pool which is part of the larger atmospheric circulation system over the North Atlantic. In fact, from 1980 to 2019, the COM of the WSC maximum lower bound has shown a possible shift northward (Fig. 5B, trend line, p = 0.13) by about 40 km and westward by about 40 km (not shown here, see Gifford^41,42^). This indicative northwestward movement of the curl maximum is similar to that observed in the decadal analysis of the GS path movement in recent decades^39^, providing further evidence of synchronicity between the two. Figure 5C shows that the COM has become more localized to a focused region, leading to the higher density towards the center of its kernel density estimation (KDE). KDE is a representation of the probability density estimator, when kernel smoothing is applied based on weights and used for finite data samples^46,47^. The red and green shaded contours in Fig. 5C represent the COMs in the recent decade, indicating agreement with Seidov et al.^39^.

## Discussion

### Reconciling theory with observations

The overall Gulf Stream system reaches from Florida to the Grand Banks and beyond, and different sub-regions within this system exhibit distinct controlling dynamics These connected sub-regions comprise (1) the attached western boundary current (part of which is the Florida Current), (2) the separation region near Cape Hatteras and (3) the free jet after separation. Our results strongly indicate that variability in the path of the separated GS (i.e. the free jet) coincides with the positive maximum region of the wind stress curl at time scales from monthly, to annual, to decadal.

Western boundary intensification as observed in subtropical oceanic gyres was first explained with a simple analytical model by Stommel^48^ for a homogenous and flat-bottom ocean. The essential model elements included a sinusoidal zonal wind stress (neglecting the much smaller meridional component), latitudinal variation of the Coriolis parameter (the beta term) and a bottom drag proportional to velocity. This formulation led to an elegant “single-gyre” stream function solution where the streamlines crowded along the western boundary in the presence of a non-zero Coriolis parameter (beta) to form a narrow, swift poleward-flowing western boundary current (see Vallis^49^, their Fig. 14.4). By construct, the sinusoidal (sin πy/b, where b is the domain width in the y-direction) zonal wind stress led to another sinusoidal function (cos πy/b) to describe the associated wind stress curl such that the zero wind stress curl lines occurred along the northern and southern boundaries of the gyre and a negative maximum curl was found along the center of the domain, where the poleward western boundary current transport was maximum.

The existence of the positive vorticity pool and the positive maximum curl within the subtropical gyres (in both the North Atlantic and North Pacific) was recognized around the same time^50,51^ from observed wind fields. Early studies of the wind-driven circulation^49,52,53^ considered linear dynamics and did not examine the dynamics within a separated boundary current like the Gulf Stream east of Cape Hatteras. Non-linear effects alter the simple picture, and the early studies were followed with “double-gyre” formulations^54,55^ and multiple numerical solutions^52,53,56^. Later, Verron and Le Provost^57^ maintained that the separation of the boundary current is largely independent of the location of the zero of the wind stress curl. A recent study covering multiple decades of observation (Figs. 3 and 4 of Seidov et al.^39^) shows the zero of the wind stress curl follows the GS path in the “extension region” (i.e., downstream of Cape Hatteras) only around 50–40 W (i.e., east of Grand Banks). In fact, the separation latitude has been shown to depend on the balance of large-scale Ekman pumping and the boundary current’s transport while following the coast by Gangopadhyay et al.^27^ based on earlier works by Parsons^58^ and Veronis^59^. This separation mechanism hypothesis was recently validated using forty years of observations^60^ and was used to develop a GS separation latitude forecasting model. In contrast to these studies of the “attached” GS, our study here focuses on the path of the GS after separation—in the 75°–55° W region, east of the separation latitude, where the dynamics of the path might be better controlled by the maximum positive vorticity pool of the wind stress as previously envisaged by several investigators^52–57^. In this study, we do not address the region further to the east of 50° W, where the mean GS path, the maximum of the WSC and the zero of the WSC, all collapse into a confined area (Figs. 3 and 4 of Seidov et al.^39^).

The amplitude of the meandering of the GS path after separation is also linked with its separation near Cape Hatteras (75° W, 35° N). The separation of the GS from the continental slope near Cape Hatteras is governed by multiple factors that include inertial control^61^, time-integrated basin-wide wind stress^26–28,58,59^ and bathymetric control^62,63^. Many studies have proposed that the path of the GS is also influenced by the southward flow of cold, fresh waters of the Labrador Current^64^, dictated by the strength and location of one of the NAO's “centers-of-action,” namely, the Icelandic low-pressure center^65,66^. A recent study by Silver et al.^60^ created a regression prediction method to forecast the GS North Wall position with 1-year lead using the Icelandic low center pressure and longitude paired with the Southern Oscillation Index (SOI). The separation of the GS from the continental margin has been discussed by Pickart and Smethie^67^ in the context of the Deep Western Boundary Current, which the GS crosses near Cape Hatteras. This might play a second order role in determining the position of the GS, especially locally just downstream of the GS separation point, and may add some of the “noise” to the robust relationship found here between wind stress curl maximum and GS position—especially at interannual scales.

The variability of the path and transport of heat and mass by the GS is also linked to the variability of the AMOC since the GS carries both wind-driven and density-driven (thermohaline) components (e.g., Bryden^68^) that are associated with horizontal and vertical recirculation cells, respectively. The AMOC transports warm limb waters northward within the upper ocean and colder, denser waters southward at depth. Since the GS carries all of the AMOC warm limb flow^69^, understanding GS path variability as a component of AMOC, might lead to a better understanding and prediction of the variability of the overall AMOC system^70,71^. As shown by Silver et al.^60^, the Parsons–Veronis two-layer model whereby Ekman wind drift affects the GS separation latitude^27,57,58^ has worked well (i.e., has good prediction skill) for four decades on the backdrop of an actively varying AMOC. Given that most of the AMOC variability is in fact dominated by this Ekman Drift^70–74^, it is possible that one could develop an AMOC predictability scheme using climate projections of the wind field with a focus on changes in the region of the maximum wind stress curl.

### Biological implications

The interplay between the region of positive wind stress curl with the GS and its meanders and WCRs has implications for biological changes near the GS in the domain of interest. This also suggests significant implications for the New England fisheries sector as well as the region’s economy and ecological health. This relationship between GS path and the positive vorticity pool demonstrated above hints at a relationship between the free GS jet and wind induced gyres and secondary circulations. This may inform understanding of the biological productivity related to upwelling regions (found beneath positive wind stress curl) versus downwelling regions (found beneath negative wind stress curl) and the ecology of the free jet region. A positive vorticity region of the wind stress curl would typically be a favorable region for positive vertical velocities^75,76^. Thus, according to the observational evidence presented here, the GS path and its latitudinal width of 150 km should be subjected to continuous upward motion due to the overlying positive wind stress curl. Multidecadal evolution of the wind field and shifts in the regions of wind stress curl maximum in the domain of interest (e.g., Fig. 5) suggest changes in Ekman pumping that can drive upwelling within and north of the GS. This upwelling has the potential to introduce more nutrient-rich water to the high light levels in the near-surface layer, and support biological productivity.

**Figure 6:**
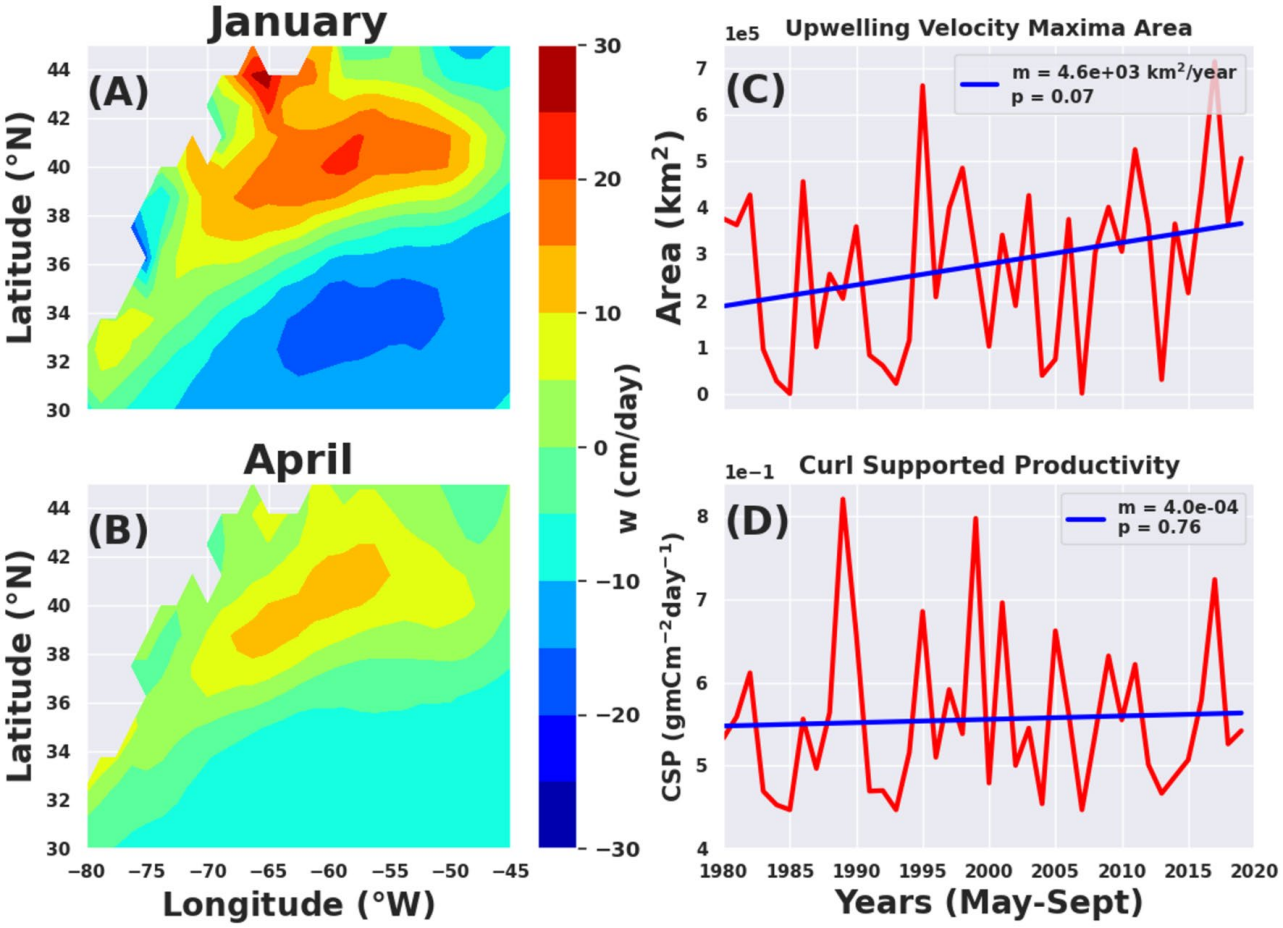
Validation of upwelling velocities and potential impact on primary productivity. (**A**) for January (maximum upwelling); (**B**) for April (springtime upwelling). The fields reasonably agree with the results of Risen and Chelton^77^. (**C**) Interannual variability of the Area covered by the maximum upwelling velocity region defined by the area bounded by the threshold of 6 cm day^−1^, which is very similar to the positive vorticity pool area. The area expansion is really high in 1995 during the nutrient limited period (May–September). (**D**) The inter-annual variability of potential wind-stress curl supported productivity (CSP in gm C m^−2^ day^−1^) averaged for the biologically active season of May–September over the positive vorticity pool. There is no significant trend in the CSP’s interannual variation.

Using a climatology of the wind field, a first order estimate of upwelling within the maximum curl region (i.e., in the positive vorticity pool) ranges from 5 cm day^−1^ (0.05 m day^−1^) in July to about 17 cm day^−1^ (0.17 m day^−1^) in January (Fig. 6A). The springtime upwelling with a peak of about 10 cm day^−1^ is also shown in Fig. 6B. This matches reasonably well with previous estimates of 5–20 cm day^−1^ seasonal fluctuations from a QuickSCAT wind analysis by Risen and Chelton^77^. These vertical velocities are relatively low, but they may be important biologically as the upwelling is continuous and occurs over a large area (400,000 km^2^) spanned by the positive maximum of the wind stress curl. If we assume a ~ 10 μM nitrate endmember at the nutricline at 50 m depth in the Slope Sea^78^, daily WSC-driven nitrate flux into the euphotic zone over the course of a season can be estimated (see methods for details). Assuming an area integrated upwelling flux of about 0.35 Sv (see Fig. 6C for area and Gifford^41^ for integrated upwelling flux) over the maximum curl region, this wind stress curl-driven upwelling would potentially provide an additional flux of 3.02 × 10^8^ mol N/day. This has the potential to support more than 24,000 tonnes C day^−1^ of primary production over the GS and Slope Sea region. When considered over the 400,000 km^2^ maximum wind stress curl region, this nitrate flux translates to about 60 mg C m^−2^ day^−1^ of primary productivity. The interannual variability in WSC-driven productivity over the period 1993–2019 is presented in Fig. 6D. It is curious to note that there is large interannual variability without any significant long-term trend in the curl supported productivity (Fig. 6D), despite the observed significant long-term trends in the positive vorticity pool area and the GS path discussed above. This lack of significant long-term trend in curl-supported productivity, despite the fact that there appears to be a clear and more statistically significant trend in upwelling velocity max area as shown in Fig. 6C, might be due to other factors including unresolved strength of upwelling from inadequate mixed-layer depth data, unknown vertical structure of upwelling, unknown distribution of rings and upwelling filaments in the slope sea underneath the positive vorticity pool.

This estimate is substantial in context of multiple metrics of productivity in the region. Estimates of primary productivity (PP) in the Mid Atlantic Bight from Ma and Smith^79^ range from 200 mg C m^−2^ day^−1^ to about 1000 mg C m^-2^ d^-1^ depending on the location and timing (see their Figs. 2 and 3 and Tables 2–4 of Ma and Smith^79^) and the type of PP models (vertically resolved, estimated from ^14^C uptake, or estimate from surface variables). Thus, our estimate of as much as 60 mg C m^−2^ day^−1^ of primary productivity supported by wind-induced upwelling would be ~ 10–25% of the current estimates of offshore primary productivity in the continental slope region of the western North Atlantic. The wind-induced upwelling likely supports a larger proportion of productivity offshore than on the shelf, as the primary productivity in the slope and Gulf Stream region is generally lower.

## Conclusions

As demonstrated here, the GS is observed to lie within the region of the wind stress curl maximum or the positive vorticity pool to the north of the zero wind stress curl. For monthly averaged values, the maximum positive vorticity line is found 70 ± 30 km north of the axis of the GS (where the standard deviation represents the along-GS variability between 70° W and 55° W), while the line of zero wind stress curl is south of the GS by about 240 ± 60 km . The whole width of the Gulf Stream (~ 150 km) falls within the region of maximum wind stress curl, i.e., the positive vorticity pool (Figs. 1, 3B,C; Figs. S1, S2, S3; and Movies S1, S2) at multiple time-scales over a 27-year period (monthly, annually and over the 27-year average). Annually, the GS is always within the region of maximum curl. Evolution of the WSC maximum shows an increase in the area and in the intensity of the maximum (increasing from 80 × 10^–9^ to 140 × 10^–9^ Pa/m); specifically a significant shift in area, AIC, and the introduction of stronger WSC values of ≥ 120 × 10^–9^ Pa/m in the pentad of 1995–1999 is observed. This has important implications on the region’s fundamental physics and on the chemistry and biology of North Atlantic ecosystems. The resulting wind-induced upwelling could support as much as 60 mg C m^−2^ day^−1^ of primary productivity, explaining about 10–25% of the estimated mean primary productivity in the offshore western North Atlantic.

The observational evidence presented herein, suggests that the meandering GS (or the “free jet”) after separation adjusts to the nearby positive maximum of the wind stress curl. We note that this 27-year-long observational phenomenon is robust and is inconsistent with the paradigm that the Gulf Stream path after separation follows the isopleth of zero in the wind stress curl field where the wind stress’s vorticity is zero. Clearly, the dynamical framework underpinning this observed alignment remains to be explored in the future with models that might (or might not) build on the early, linear theories of (attached) western boundary currents. Note that it is possible that the GS path is not just passively responding to the overlying wind field. The GS is known to actively influence the wind field above it via impacts on surface winds, atmospheric convergences and divergences, rain bands, air-sea fluxes, etc.^80–82^. The implication of the synchronicity found here between the wind stress curl maximum and the position of the warm GS path over the span of 1500–2500 km opens up a range of questions related to ocean-atmosphere coupling in the western North Atlantic across temporal scales spanning storms, to weather, and to climate. This observational evidence suggests the possibility of development of new ways of understanding the behavior of the GS path as a free jet after Cape Hatteras, especially in the 75°–55° W region, which is important for studying future AMOC scenarios by more clearly delineating how Gulf Stream variability relates to changes in AMOC.

### Supplementary Information

Supplementary Information.

Supplementary Video 1.

Supplementary Video 2.

## Data Availability

The wind velocity data is freely available from the Japan Meteorological Agency’s 55-year reanalysis (JRA-55) distribution site at https://jra.kishou.go.jp/JRA-55/atlas/en/index.html. The wind stress and wind stress curl data computed for this study are available in the zenodo repository at https://zenodo.org/record/8200832. The Gulf Stream paths were extracted from altimeter gridded product available at https://www.copernicus.eu/en. The monthly and annual Gulf Stream paths, and contours of zero and maximum wind stress curl are available at https://zenodo.org/records/8217388. The Curl distribution, upwelling fields and other datasets generated or analyzed during the study are available from the corresponding author on reasonable request. All the data are available in the links provided in the Supplementary Information and in the Data availability statement. All of the data generated or analyzed during the current study are available from the corresponding author on reasonable request.
