# Supplementary material for: Synchronicity of the Gulf Stream path downstream of Cape Hatteras and the region of maximum wind stress curl
Source: Sci Rep. 2024 Aug 9;14:18479. doi: 10.1038/s41598-024-68461-0 (PMC11315899; doi:10.1038/s41598-024-68461-0)
Supplement: Supplementary file 1 — Supplementary Information. [file 41598_2024_68461_MOESM1_ESM.docx]

Supplementary Information for

**Synchronicity of the Gulf Stream Path Downstream of Cape Hatteras and the Region of Maximum Wind Stress Curl**

Ian Gifford, Avijit Gangopadhyay*, Magdalena Andres, Hilde Oliver, Glen Gawarkiewicz and Adrienne Silver

*Corresponding author: Avijit Gangopadhyay, Email: [avijit.gangopadhyay@umassd.edu](mailto:avijit.gangopadhyay@umassd.edu)

**This PDF file includes:**

Supplementary Text Supplementary figure 1

Supplementary figure 2

Supplementary figure 3 Captions for Movies S1 to S2 References (83 to 87)

**Other Supplementary Information for this manuscript include the following:**

Movies S1 to S2

**Supplementary Text**

Materials and Methods

Winds

This project mainly utilizes the Japan Meteorological Agency's 55-year reanalysis (*83*). Six- hourly surface winds in the zonal and meridional direction from 1980 to 2019 are analyzed over the region 80W to 45W and 30N to 45N. Wind velocity, stress, and wind stress curl can be accessed at <https://zenodo.org/record/8200832>. The MERRA-2 dataset (*84*), which has a higher resolution (0.625), but limited until 2010 in its quality, was explored with the same methodology as the JRA-55 data to verify results found with the latter dataset. This test also signifies the robustness of the curl field from a 1.25 dataset. See (*41*) for details. Future high-resolution winds might shed more detailed light on the synchronicity presented here.

Six-hour surface winds were processed using Python. Wind stress was computed using a formulation based on (*26*) and a non-linear drag coefficient (*85*). The expression is modified later for low speeds (*86*). Once wind stress is calculated, the zonal change in the meridional stress (Curlx) and the negative meridional change in zonal stress (Curly) were found using the central finite difference method. The total curl over the domain is calculated by summing Curlx and Curly. Various climatologies (monthly, seasonal, annual, 5-year and 27-year) are computed from the 6-hourly fields of the WSC.

PDF distribution and Curl contour determination

A PDF distribution (**Fig. 2**) of the prominence of 27-year averaged WSC values was generated to allow the quantification of WSC maxima. The pdf distribution was obtained in four steps: (i) the annual values of WSC for 27 years were chosen as the sample for the curl field; (ii) 1000 contour intervals between the most negative (-200 x 10-9 Pa/m) and the most positive (230 x 10-9 Pa/m) were then chosen for determining their individual occupancy within the study domain; (iii) a histogram of the distribution of occupancy of curl values was generated by using 32 bins (integer rounded for 1000); and (iv) the resulting histogram was normalized with the maximum occupancy of the bin (which is very close to the zero curl) to yield the final PDF in Fig. 2.

Next, the mean and standard deviation (STD) was found, and the PDF was used to find the range of wind stress curl values between 1.3 and 3.5 STD from the mean. After the maxima is applied to the domain (see Fig. 3), the areas of a spread of curl intervals 10 x 10-9 Pa/m units apart from the lower bound (80 x 10-9 Pa/m) up to the upper bound (190 x 10-9 Pa/m) was calculated using the WGS84 model with a shoelace algorithm in Python (*41*). In other words, the maxima region for each year were broken down in multiple annuluses with mean values increasing by 10 x 10-9 Pa/m starting from 80 x 10-9 Pa/m. The Area integrated curl were then the sum of individual contribution of curl from each annulus or the area of annulus weighted by the mean curl of a particular annulus.

The WSC max contour is the maximum wind stress curl value at each 0.1 degree increment longitude in the domain excluding values near the coast. Zero wind stress curl is similarly defined as the coordinates of the location of the zero curl at each longitude within the domain. For longitudes in which two locations of zero curl are present the first location of zero curl south of the max curl location was selected. Note that the monthly MCLs and ZCLs are obtained from the monthly wind-stress curl fields. The annual MCLs and ZCLs are obtained from the annual wind-stress curl fields. Since the monthly curl fields preserves more atmospheric mesoscales than the annual curl fields, the 12-month average of the monthly MCLs and ZCLs will not match with the annual MCLs and ZCLs derived from the annual curl field. The annual MCLs and ZCLs are obtained from the annual curl fields and representative metrics of the wind forcing on an annual time-scale. See (*41, 42*) for more details.

Uncertainty of JRA-55 wind data

It is known that JRA-55 winds display high correlations (>0.9) and high signal-to-noise ratio (>3) for both stress and curl fields in the mid-latitude regions of Atlantic and Pacific oceans with other reanalysis products and available buoy observations (*87*).

Gulf Stream Path Dataset

The Gulf Stream data is daily 25-cm contours generated from altimetry data following the methods described in (*5*). The GS path at monthly and annual time-scales are available from (*42*). Multivalued paths, when the 25-cm contours folds back upon itself and crosses a given longitude, have been smoothed by using the consolidator function in MATLAB for uniqueness.

Upwelling N/C flux computation

This computation is done in six steps as follows. (i) First, the maxima upwelling flux (which is the area integrated upwelling velocity) for May-Sept (see *41*) is used to consider an average volume flux of 0.35 Sverdrup or 0.35 x 106 m3/s. (ii) Second, we note that the typical nutrient flux of 10 µM N at the base of the mixed layer in this region (*78*) is equivalent to 10 millimol/m3. (iii) Third, the Nitrate flux in mmol/day is calculated as equal to: nutricline concentration (mmol N m-3) x vertical velocity (m s^-1^) x area (m2) x (86400 seconds/day) = flux in mmol/day. This leads to: 10 mmol/m3 x 0.35 x 10^6^ m3 x 86400 s/day = 3.02 x 10^8^ mol N/day as the nutrient flux. (iv) Next, we use the Redfield ratio (C/N=106/16) to convert to mol C: 2.0034 x 109 mol C d-1. (v) We then multiply by the molar mass of carbon (12.01 gm /mol) to convert to gm C: 2.4061 x 1010 gm C d-1 = 24061 tonnes C d-1 (using 1 gm = 1 x 10-6 tonnes), for the entire slope sea under the max curl region. (vi) Finally, using the average area of maximum upwelling region over the years (Fig. 6B), which is about 400,000 sq km, we arrive at a potential productivity rate supported by this wind-stress curl driven upwelling to be about 0.060 gm C m-2 d-1 or 60 mg C m-2 d-1.

**References**

- S. Kobayashi, Y. Ota, Y. Harada, A. Ebita, M. Moriya, H. Onoda, K. Onogi, H. Kamahori, C. Kobayashi, H. Endo, K. Miyaoka, The JRA-55 reanalysis: General specifications and basic characteristics. Journal of the Meteorological Society of Japan. Ser. II, 93(1), pp.5-48. (2015).
- Gelaro, R., McCarty, W., Suárez, M.J., Todling, R., Molod, A., Takacs, L., Randles, C.A., Darmenov, A., Bosilovich, M.G., Reichle, R. and Wargan, K., 2017. The modern-era retrospective analysis for research and applications, version 2 (MERRA-2). *Journal of climate*, *30*(14), pp.5419-5454.
- W.G. Large, S. Pond, 1981. Open ocean momentum flux measurements in moderate to strong winds. Journal of physical oceanography, 11(3), pp.324-336 (1981).
- K.E. Trenberth, W.G. Large, J.G. Olson, The effective drag coefficient for evaluating wind stress over the oceans. Journal of Climate, 2(12), pp.1507-1516 (1989).
- C. Wen, A. Kumar, Y. Xue, Uncertainties in reanalysis surface wind stress and their relationship with observing systems. Climate Dynamics, 52, pp.3061-3078 (2019).

**Supplementary figure 1**

**Annual WSC and GS superimposed for each year during the altimetry era (1993-2019).** Note the consistency of the free jet lying within the region of maximum of the wind stress curl on an annual basis. The red line is line of annual maxima of the wind stress curl (MCL) and the green thick line is the annual GS path for each year. The zero WSC contour (ZCL, blue) is marked with a zero. Negative WSC contours (dashed) extend from -200 to -50 Pa/m x 10^-9^ with intervals of 50 Pa/m x 10^-9^. Color scheme is similar to that in Fig. 1 in the main text.

**Supplementary figure 2**

**Synchronicity on monthly scale.** Average monthly wind stress curl field superimposed with average monthly GS path based on 27-years of reanalysis and altimetry data. Curl amplitude values are shown in contours of (x 10^-9^ Pa/m).

**Supplementary figure 3**

**Synchronicity on seasonal scale.** Average seasonal wind stress curl field superimposed with average seasonal GS path based on 27-years of reanalysis and altimetry data. Curl amplitude values are shown in contours of (x 10^-9^ Pa/m).

**Movie S1.**

This animation displays the annually averaged Wind Stress Curl (WSC) (contours) along with the corresponding annual Gulf Stream (GS) path (light blue line) from altimetry, over the domain of 80 to 45 W and 30 to 45 N. Curl amplitude values are represented in contours of (x 10^-9^ Pa/m). The animation covers 27 years of annual fields from 1993 through 2019. The shaded orange regions indicate the ‘positive vorticity pool’, defined by the region of maximum wind stress curl (see text). The light blue ‘envelope’ shows the latitudinal spread of the 1993-2019 annual mean GS paths, calculated at each 0.1° longitude bin. Additionally the animation includes the maximum Curl Line (MCL) (red solid line), Zero Curl Line (ZCL) (thin blue line), and the GS path (light blue) for each year over the 27-year period. Notably, the proximity of the MCL to the GS path is consistent each year. The GS traverses the region of maximum wind stress curl throughout all 27 years, highlighting the temporal and spatial synchronicities between the two as the animation progresses from year to year.

**Movie S2.**

This animation shows similar fields as in Movie S1 but for every month during the Altimetry period January 1993 through December 2019. A total of 324 months. Note that while the mesoscale variability of both the wind field and the Gulf Stream are much greater in the monthly fields, the overall synchronicity of the GS path and the region of maximum curl or the positive vorticity pool is apparent in most of the months over all the years. Curl amplitude values are shown in contours of (x 10^-9^ Pa/m).
